# [8-(4-But­oxy­benzo­yl)-2,7-dimeth­oxy­naphthalen-1-yl](4-but­oxy­phen­yl)methanone

**DOI:** 10.1107/S1600536811048550

**Published:** 2011-11-19

**Authors:** Kosuke Sasagawa, Toyokazu Muto, Akiko Okamoto, Hideaki Oike, Noriyuki Yonezawa

**Affiliations:** a Department of Organic and Polymer Materials Chemistry, Tokyo University of Agriculture & Technology, Koganei, Tokyo 184-8588, Japan

## Abstract

The mol­ecule of the title compound, C_34_H_36_O_6_, is located on a twofold rotation axis. The two 4-but­oxy­benzoyl groups at the 1- and 8-positions of the naphthalene ring system are aligned almost anti­parallel. The dihedral angles between the benzene rings and the naphthalene ring system are 71.70 (4)°. In the crystal, the mol­ecules are connected *via* C—H⋯π inter­actions into a layer parallel to (010).

## Related literature

For electrophilic aromatic aroylation of the naphthalene core, see: Okamoto & Yonezawa (2009 ▶); Okamoto *et al.* (2011 ▶). For the structures of closely related compounds, see: Hijikata *et al.* (2010 ▶); Muto *et al.* (2010 ▶); Nakaema *et al.* (2008 ▶); Watanabe *et al.* (2010 ▶); Sasagawa *et al.* (2011 ▶).
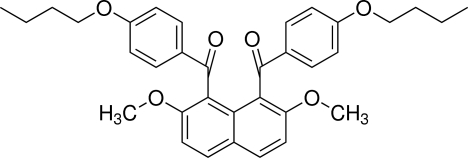


## Experimental

### 

#### Crystal data

C_34_H_36_O_6_
*M* *_r_* = 540.63
Orthorhombic, 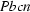

*a* = 11.0930 (2) Å
*b* = 20.0537 (3) Å
*c* = 13.1409 (2) Å
*V* = 2923.26 (8) Å^3^
*Z* = 4
Cu *K*α radiation
μ = 0.67 mm^−1^
*T* = 193 K
0.60 × 0.40 × 0.20 mm

#### Data collection

Rigaku R-AXIS RAPID diffractometer
Absorption correction: numerical (*NUMABS*; Higashi, 1999 ▶) *T*_min_ = 0.689, *T*_max_ = 0.878
50625 measured reflections
2679 independent reflections
2542 reflections with *I* > 2σ(*I*)
*R*_int_ = 0.028

#### Refinement

*R*[*F* ^2^ > 2σ(*F* ^2^)] = 0.037
*wR*(*F* ^2^) = 0.105
*S* = 1.04
2679 reflections
185 parameters
H-atom parameters constrained
Δρ_max_ = 0.27 e Å^−3^
Δρ_min_ = −0.16 e Å^−3^

### 

Data collection: *PROCESS-AUTO* (Rigaku, 1998 ▶); cell refinement: *PROCESS-AUTO*; data reduction: *CrystalStructure* (Rigaku, 2010 ▶); program(s) used to solve structure: *SIR2004* (Burla *et al.*, 2005 ▶); program(s) used to refine structure: *SHELXL97* (Sheldrick, 2008 ▶); molecular graphics: *ORTEPIII* (Burnett & Johnson, 1996 ▶); software used to prepare material for publication: *SHELXL97*.

## Supplementary Material

Crystal structure: contains datablock(s) 1_8-obu-shelxl, global, I. DOI: 10.1107/S1600536811048550/gk2430sup1.cif

Structure factors: contains datablock(s) I. DOI: 10.1107/S1600536811048550/gk2430Isup2.hkl

Supplementary material file. DOI: 10.1107/S1600536811048550/gk2430Isup3.cml

Additional supplementary materials:  crystallographic information; 3D view; checkCIF report

## Figures and Tables

**Table 1:** Hydrogen-bond geometry (Å, °) *Cg* is the centroid of the C9–C14 ring.

*D*—H⋯*A*	*D*—H	H⋯*A*	*D*⋯*A*	*D*—H⋯*A*
C7—H7*A*⋯*Cg*^i^	0.98	2.68	3.5056 (14)	142

Symmetry code: (i) 
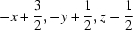
.

**Fig. 1.**
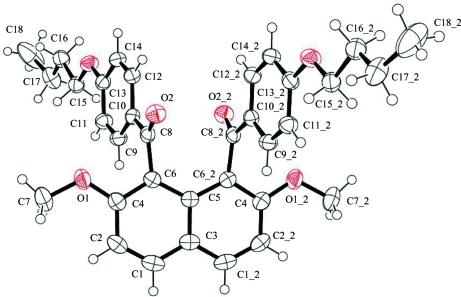
Molecular structure with displacement ellipsoids drawn at the 50% probability level. The symbol "_2" refers to symmetry code: -x+1, y, -z+1/2.

**Fig. 2.**
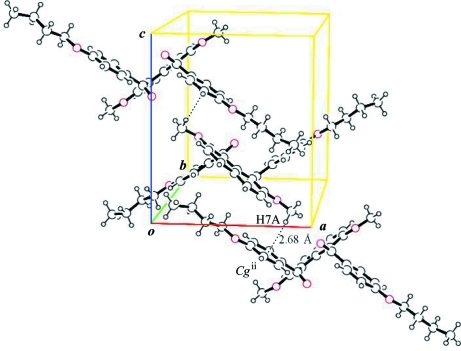
Intermolecular C-H···π interactions (dashed lines). Cg is the centroid of the C9–C14 ring [symmetry code: (ii) -x+3/2, -y+1/2, z-1/2].

## supplementary crystallographic information

### Comment

In the course of our study on electrophilic aromatic aroylation of the naphthalene core, 1,8-diaroylnaphthalene compounds have proved to be formed regioselectively by the aid of a suitable acidic mediator (Okamoto & Yonezawa, 2009, Okamoto *et al.*, 2011). Recently, we have reported the X-ray crystal structures of 1,8-diaroylated 2,7-dimethoxynaphthalene derivatives such as 1,8-dibenzoyl-2,7-dimethoxynaphthalene (Nakaema *et al.*, 2008), (2,7-dimethoxynaphthalene-1,8-diyl)bis(4-fluorophenyl)dimethanone [1,8-bis(4-fluorobenzoyl)-2,7-dimethoxynaphthalene] (Watanabe, Nagasawa *et al.*, 2010), 1,8-bis(4-methylbenzoyl)-2,7-dimethoxynaphthalene (Muto *et al.*, 2010), and {8-[4-(bromomethyl)benzoyl]-2,7-dimethoxynaphthalen-1-yl}[4-(bromomethyl)phenyl]methanone [1,8-bis(4-bromomethylbenzoyl)-2,7-dimethoxynaphthalene] (Sasagawa *et al.*, 2011). The aroyl groups in these compounds are perpendicularly attached to the naphthalene rings and oriented in opposite directions. On the other hand, X-ray structure of 2,7-dimethoxy-1,8-bis(4-phenoxybenzoyl)naphthalene (Hijikata *et al.*, 2010) having the aroyl groups oriented in the same directions has been also revealed. As a part of our ongoing studies on the molecular structures of this kind of homologous molecules, the X-ray crystal structure of title compound, 1,8-diaroylnaphthalene bearing butoxy groups, is discussed in this article.

The molecular structure of the title compound is displayed in Fig 1. The molecule of (I) lies on a crystallographic 2-fold axis so that the asymmetric unit contains one-half of the molecule. Thus, two 4-butoxybenzoyl groups are situated in anti orientation and are twisted away from the attached naphthalene ring. The dihedral angle between the best planes of the 4-butoxyphenyl groups and the naphthalene ring system is 71.70 (4)°.

The dihedral between the naphthalene ring system and the bridging carbonyl C—C(═O)—C plane is 77.60 (5)° [C5—C6—C8—O2 torsion angle = -77.75 (12)°], far larger than that [8.64 (5)°; C12—C10—C8—O2 torsion angle = 8.33 (14)°] between the phenyl group and the bridging carbonyl group.

In the crystal, molecules are arranged into (0 1 0) layers via C-H···π interactions (Fig. 2).

### Experimental

The title compound was prepared by S_N_2 reaction of 1,8-bis(4-hydroxybenzoyl)-2,7-dimethoxynaphthalene (1.0 mmol, 428.5 mg), which was obtained *via* S_N_Ar reaction of 1,8-bis(4-fluorobenzoyl)-2,7-dimethoxynaphthalene with sodium hydroxide, with bromobutane (3.0 mmol, 411 mg) and potassium carbonate (2.8 mmol, 387 mg) in *N*,*N*-dimethylformamide (DMF; 2.5 ml). After the reaction mixture was stirred at 333 K for 6 h, it was poured into water (30 ml) and the mixture was extracted with CHCl_3_ (15 ml × 3). The combined extracts were washed with brine. The organic layers thus obtained were dried over anhydrous MgSO_4_. The solvent was removed under reduced pressure to give cake (96% yield). The crude product was purified by recrystallization from methanol (isolated yield 65%). Furthermore, the isolated product was crystallized from methanol to give single crystal. Spectroscopic data:^1^H NMR δ(300 MHz, CDCl_3_); 0.98(6*H*, t, *J* = 7.2), 1.49(4*H*, m, *J* = 7.5 Hz), 1.77(4*H*, q, *J* = 8.1 Hz), 3.70(6*H*, s), 3.98(4*H*, m), 6.80(4*H*, broad), 7.20(2*H*, d, *J* = 9.0 Hz), 7.65(4*H*, broad), 7.92(2*H*, d, *J* = 9.3 Hz) p.p.m.. ^13^C NMR δ(100 MHz, CDCl_3_); 13.8, 19.2, 31.2, 56.5, 67.7, 111.3, 113.4, 122.0, 125.6, 129.6, 131.4, 131.6, 131.8, 155.9, 162.7, 194.9 p.p.m.. IR (KBr); 2956, 2936, 1665, 1600, 1509, 1267, 1250 cm^-1^. (m/*z*): [*M* + H]^+^ Calcd for C_34_H_37_O_6_, 541.2590; found, 541.2559. m.p. = 392–399.9 K

### Refinement

All H atoms were found in a difference map and were subsequently refined as riding atoms, with C—H = 0.95 -0.99 Å, and with *U*_ĩso_(H) = 1.2 *U*_eq_(C).

### Crystal data

**Table:**

C_34_H_36_O_6_	*F*(000) = 1152
*M**_r_* = 540.63	*D*_x_ = 1.228 Mg m^−^^3^
Orthorhombic, *P**b**c**n*	Cu *K*α radiation, λ = 1.54187 Å
Hall symbol: -P 2n 2ab	Cell parameters from 47089 reflections
*a* = 11.0930 (2) Å	θ = 3.4–68.2°
*b* = 20.0537 (3) Å	µ = 0.67 mm^−^^1^
*c* = 13.1409 (2) Å	*T* = 193 K
*V* = 2923.26 (8) Å^3^	Block, colorless
*Z* = 4	0.60 × 0.40 × 0.20 mm

### Data collection

**Table:**

Rigaku R-AXIS RAPID diffractometer	2679 independent reflections
Radiation source: fine-focus sealed tube	2542 reflections with *I* > 2σ(*I*)
graphite	*R*_int_ = 0.028
Detector resolution: 10.000 pixels mm^-1^	θ_max_ = 68.2°, θ_min_ = 4.4°
ω scans	*h* = −13→13
Absorption correction: numerical (*NUMABS*; Higashi, 1999)	*k* = −24→24
*T*_min_ = 0.689, *T*_max_ = 0.878	*l* = −15→15
50625 measured reflections	

### Refinement

**Table:**

Refinement on *F*^2^	Secondary atom site location: difference Fourier map
Least-squares matrix: full	Hydrogen site location: inferred from neighbouring sites
*R*[*F*^2^ > 2σ(*F*^2^)] = 0.037	H-atom parameters constrained
*wR*(*F*^2^) = 0.105	*w* = 1/[σ^2^(*F*_o_^2^) + (0.0608*P*)^2^ + 0.5804*P*] where *P* = (*F*_o_^2^ + 2*F*_c_^2^)/3
*S* = 1.04	(Δ/σ)_max_ < 0.001
2679 reflections	Δρ_max_ = 0.27 e Å^−^^3^
185 parameters	Δρ_min_ = −0.16 e Å^−^^3^
0 restraints	Extinction correction: *SHELXL97* (Sheldrick, 2008), Fc^*^=kFc[1+0.001xFc^2^λ^3^/sin(2θ)]^-1/4^
Primary atom site location: structure-invariant direct methods	Extinction coefficient: 0.0027 (2)

### Special details

**Table:**

Geometry. All e.s.d.'s (except the e.s.d. in the dihedral angle between two l.s. planes) are estimated using the full covariance matrix. The cell e.s.d.'s are taken into account individually in the estimation of e.s.d.'s in distances, angles and torsion angles; correlations between e.s.d.'s in cell parameters are only used when they are defined by crystal symmetry. An approximate (isotropic) treatment of cell e.s.d.'s is used for estimating e.s.d.'s involving l.s. planes.
Refinement. Refinement of *F*^2^ against ALL reflections. The weighted *R*-factor *wR* and goodness of fit *S* are based on *F*^2^, conventional *R*-factors *R* are based on *F*, with *F* set to zero for negative *F*^2^. The threshold expression of *F*^2^ > σ(*F*^2^) is used only for calculating *R*-factors(gt) *etc*. and is not relevant to the choice of reflections for refinement. *R*-factors based on *F*^2^ are statistically about twice as large as those based on *F*, and *R*- factors based on ALL data will be even larger.

### Fractional atomic coordinates and isotropic or equivalent isotropic displacement parameters (Å^2^)

**Table:**

	*x*	*y*	*z*	*U*_iso_*/*U*_eq_	
O1	0.75396 (7)	0.21648 (4)	0.08148 (6)	0.0440 (2)	
O2	0.54194 (7)	0.32529 (4)	0.13838 (6)	0.0393 (2)	
O3	0.98869 (7)	0.41391 (4)	0.40338 (6)	0.0393 (2)	
C1	0.58364 (10)	0.07697 (5)	0.18871 (8)	0.0412 (3)	
H1	0.5810	0.0296	0.1871	0.049*	
C2	0.66800 (10)	0.10911 (6)	0.13181 (9)	0.0413 (3)	
H2	0.7227	0.0847	0.0906	0.050*	
C3	0.5000	0.11152 (7)	0.2500	0.0353 (3)	
C4	0.67285 (10)	0.17957 (5)	0.13508 (8)	0.0357 (3)	
C5	0.5000	0.18316 (7)	0.2500	0.0301 (3)	
C6	0.59200 (9)	0.21598 (5)	0.19277 (7)	0.0308 (2)	
C7	0.85144 (11)	0.18354 (7)	0.03189 (10)	0.0516 (3)	
H7A	0.8196	0.1527	−0.0193	0.062*	
H7B	0.8987	0.1586	0.0822	0.062*	
H7C	0.9031	0.2167	−0.0013	0.062*	
C8	0.60813 (9)	0.29099 (5)	0.19033 (7)	0.0304 (2)	
C9	0.79477 (9)	0.28188 (5)	0.29938 (8)	0.0343 (3)	
H9	0.7888	0.2347	0.2965	0.041*	
C10	0.70756 (9)	0.32031 (5)	0.25103 (7)	0.0303 (2)	
C11	0.89010 (10)	0.31051 (5)	0.35162 (8)	0.0361 (3)	
H11	0.9489	0.2833	0.3837	0.043*	
C12	0.71586 (9)	0.38984 (5)	0.25911 (8)	0.0339 (3)	
H12	0.6561	0.4171	0.2283	0.041*	
C13	0.89867 (9)	0.37990 (5)	0.35653 (8)	0.0335 (2)	
C14	0.80960 (10)	0.41894 (5)	0.31120 (8)	0.0356 (3)	
H14	0.8137	0.4661	0.3164	0.043*	
C15	1.08084 (9)	0.37643 (5)	0.45496 (8)	0.0372 (3)	
H15A	1.1186	0.3443	0.4075	0.045*	
H15B	1.0454	0.3512	0.5124	0.045*	
C16	1.17371 (10)	0.42488 (6)	0.49380 (9)	0.0426 (3)	
H16A	1.1332	0.4600	0.5342	0.051*	
H16B	1.2141	0.4466	0.4354	0.051*	
C17	1.26786 (11)	0.39032 (8)	0.55954 (10)	0.0554 (4)	
H17A	1.2301	0.3767	0.6246	0.067*	
H17B	1.2955	0.3494	0.5244	0.067*	
C18	1.37590 (14)	0.43398 (11)	0.58200 (19)	0.0991 (8)	
H18A	1.4270	0.4122	0.6329	0.119*	
H18B	1.3484	0.4771	0.6083	0.119*	
H18C	1.4222	0.4409	0.5194	0.119*	

### Atomic displacement parameters (Å^2^)

**Table:**

	*U*^11^	*U*^22^	*U*^33^	*U*^12^	*U*^13^	*U*^23^
O1	0.0409 (4)	0.0484 (5)	0.0426 (4)	0.0008 (3)	0.0116 (3)	−0.0090 (3)
O2	0.0404 (4)	0.0392 (4)	0.0384 (4)	0.0020 (3)	−0.0056 (3)	0.0044 (3)
O3	0.0378 (4)	0.0335 (4)	0.0464 (5)	−0.0054 (3)	−0.0045 (3)	−0.0031 (3)
C1	0.0504 (7)	0.0292 (5)	0.0441 (6)	0.0062 (4)	−0.0142 (5)	−0.0048 (4)
C2	0.0433 (6)	0.0391 (6)	0.0415 (6)	0.0099 (5)	−0.0049 (5)	−0.0110 (5)
C3	0.0404 (8)	0.0297 (7)	0.0357 (7)	0.000	−0.0121 (6)	0.000
C4	0.0349 (6)	0.0395 (6)	0.0326 (5)	0.0017 (4)	−0.0035 (4)	−0.0062 (4)
C5	0.0314 (7)	0.0305 (7)	0.0285 (7)	0.000	−0.0068 (5)	0.000
C6	0.0313 (5)	0.0319 (5)	0.0292 (5)	0.0001 (4)	−0.0045 (4)	−0.0031 (4)
C7	0.0383 (6)	0.0677 (8)	0.0489 (7)	0.0064 (6)	0.0056 (5)	−0.0180 (6)
C8	0.0309 (5)	0.0337 (5)	0.0266 (5)	0.0006 (4)	0.0048 (4)	0.0007 (4)
C9	0.0373 (5)	0.0274 (5)	0.0381 (6)	−0.0016 (4)	−0.0001 (4)	0.0000 (4)
C10	0.0315 (5)	0.0310 (5)	0.0285 (5)	−0.0015 (4)	0.0046 (4)	0.0011 (4)
C11	0.0351 (5)	0.0326 (5)	0.0406 (6)	0.0006 (4)	−0.0039 (4)	0.0012 (4)
C12	0.0359 (5)	0.0316 (5)	0.0341 (5)	0.0012 (4)	0.0024 (4)	0.0030 (4)
C13	0.0336 (5)	0.0338 (5)	0.0331 (5)	−0.0054 (4)	0.0037 (4)	−0.0029 (4)
C14	0.0407 (6)	0.0270 (5)	0.0392 (6)	−0.0026 (4)	0.0038 (4)	0.0005 (4)
C15	0.0344 (5)	0.0382 (6)	0.0392 (6)	−0.0016 (4)	0.0018 (4)	−0.0031 (4)
C16	0.0382 (6)	0.0439 (6)	0.0456 (6)	−0.0028 (5)	0.0004 (5)	−0.0104 (5)
C17	0.0433 (7)	0.0710 (9)	0.0520 (7)	0.0103 (6)	−0.0045 (6)	−0.0159 (6)
C18	0.0470 (8)	0.1050 (14)	0.1453 (18)	0.0234 (9)	−0.0338 (10)	−0.0661 (13)

### Geometric parameters (Å, °)

**Table:**

O1—C4	1.3615 (13)	C9—C10	1.3906 (14)
O1—C7	1.4249 (13)	C9—H9	0.9500
O2—C8	1.2159 (12)	C10—C12	1.4012 (14)
O3—C13	1.3570 (12)	C11—C13	1.3963 (14)
O3—C15	1.4384 (13)	C11—H11	0.9500
C1—C2	1.3602 (16)	C12—C14	1.3750 (15)
C1—C3	1.4106 (13)	C12—H12	0.9500
C1—H1	0.9500	C13—C14	1.3942 (15)
C2—C4	1.4146 (16)	C14—H14	0.9500
C2—H2	0.9500	C15—C16	1.5052 (15)
C3—C1^i^	1.4106 (13)	C15—H15A	0.9900
C3—C5	1.437 (2)	C15—H15B	0.9900
C4—C6	1.3828 (14)	C16—C17	1.5222 (17)
C5—C6	1.4284 (12)	C16—H16A	0.9900
C5—C6^i^	1.4284 (12)	C16—H16B	0.9900
C6—C8	1.5151 (14)	C17—C18	1.513 (2)
C7—H7A	0.9800	C17—H17A	0.9900
C7—H7B	0.9800	C17—H17B	0.9900
C7—H7C	0.9800	C18—H18A	0.9800
C8—C10	1.4828 (14)	C18—H18B	0.9800
C9—C11	1.3853 (14)	C18—H18C	0.9800
			
C4—O1—C7	119.09 (10)	C9—C11—H11	120.4
C13—O3—C15	118.31 (8)	C13—C11—H11	120.4
C2—C1—C3	122.25 (10)	C14—C12—C10	120.65 (10)
C2—C1—H1	118.9	C14—C12—H12	119.7
C3—C1—H1	118.9	C10—C12—H12	119.7
C1—C2—C4	118.87 (10)	O3—C13—C14	115.67 (9)
C1—C2—H2	120.6	O3—C13—C11	124.88 (10)
C4—C2—H2	120.6	C14—C13—C11	119.45 (9)
C1^i^—C3—C1	121.15 (13)	C12—C14—C13	120.68 (9)
C1^i^—C3—C5	119.42 (7)	C12—C14—H14	119.7
C1—C3—C5	119.42 (7)	C13—C14—H14	119.7
O1—C4—C6	115.16 (9)	O3—C15—C16	108.00 (9)
O1—C4—C2	123.54 (10)	O3—C15—H15A	110.1
C6—C4—C2	121.29 (10)	C16—C15—H15A	110.1
C6—C5—C6^i^	125.13 (13)	O3—C15—H15B	110.1
C6—C5—C3	117.44 (6)	C16—C15—H15B	110.1
C6^i^—C5—C3	117.44 (6)	H15A—C15—H15B	108.4
C4—C6—C5	120.58 (10)	C15—C16—C17	111.61 (10)
C4—C6—C8	115.85 (9)	C15—C16—H16A	109.3
C5—C6—C8	123.57 (9)	C17—C16—H16A	109.3
O1—C7—H7A	109.5	C15—C16—H16B	109.3
O1—C7—H7B	109.5	C17—C16—H16B	109.3
H7A—C7—H7B	109.5	H16A—C16—H16B	108.0
O1—C7—H7C	109.5	C18—C17—C16	113.00 (14)
H7A—C7—H7C	109.5	C18—C17—H17A	109.0
H7B—C7—H7C	109.5	C16—C17—H17A	109.0
O2—C8—C10	121.79 (9)	C18—C17—H17B	109.0
O2—C8—C6	120.15 (9)	C16—C17—H17B	109.0
C10—C8—C6	118.05 (8)	H17A—C17—H17B	107.8
C11—C9—C10	121.85 (9)	C17—C18—H18A	109.5
C11—C9—H9	119.1	C17—C18—H18B	109.5
C10—C9—H9	119.1	H18A—C18—H18B	109.5
C9—C10—C12	118.11 (9)	C17—C18—H18C	109.5
C9—C10—C8	122.92 (9)	H18A—C18—H18C	109.5
C12—C10—C8	118.97 (9)	H18B—C18—H18C	109.5
C9—C11—C13	119.21 (10)		
			
C3—C1—C2—C4	0.70 (15)	C4—C6—C8—C10	−76.66 (11)
C2—C1—C3—C1^i^	−177.57 (11)	C5—C6—C8—C10	103.44 (10)
C2—C1—C3—C5	2.43 (11)	C11—C9—C10—C12	−2.11 (15)
C7—O1—C4—C6	170.91 (9)	C11—C9—C10—C8	176.85 (9)
C7—O1—C4—C2	−10.34 (15)	O2—C8—C10—C9	−170.62 (9)
C1—C2—C4—O1	179.73 (9)	C6—C8—C10—C9	8.16 (14)
C1—C2—C4—C6	−1.59 (16)	O2—C8—C10—C12	8.33 (14)
C1^i^—C3—C5—C6	175.43 (7)	C6—C8—C10—C12	−172.88 (9)
C1—C3—C5—C6	−4.57 (7)	C10—C9—C11—C13	0.42 (16)
C1^i^—C3—C5—C6^i^	−4.57 (7)	C9—C10—C12—C14	1.70 (15)
C1—C3—C5—C6^i^	175.43 (7)	C8—C10—C12—C14	−177.30 (9)
O1—C4—C6—C5	178.06 (7)	C15—O3—C13—C14	177.64 (9)
C2—C4—C6—C5	−0.73 (14)	C15—O3—C13—C11	−2.08 (14)
O1—C4—C6—C8	−1.84 (13)	C9—C11—C13—O3	−178.58 (9)
C2—C4—C6—C8	179.37 (9)	C9—C11—C13—C14	1.70 (15)
C6^i^—C5—C6—C4	−176.24 (10)	C10—C12—C14—C13	0.38 (15)
C3—C5—C6—C4	3.76 (10)	O3—C13—C14—C12	178.15 (9)
C6^i^—C5—C6—C8	3.65 (7)	C11—C13—C14—C12	−2.11 (15)
C3—C5—C6—C8	−176.35 (7)	C13—O3—C15—C16	176.09 (9)
C4—C6—C8—O2	102.15 (11)	O3—C15—C16—C17	173.78 (9)
C5—C6—C8—O2	−77.75 (12)	C15—C16—C17—C18	168.20 (12)

Symmetry codes: (i) −*x*+1, *y*, −*z*+1/2.

### Hydrogen-bond geometry (Å, °)

**Table:**

Cg is the centroid of the C9–C14 ring.

**Table:**

*D*—H···*A*	*D*—H	H···*A*	*D*···*A*	*D*—H···*A*
C7—H7A···Cg^ii^	0.98	2.68	3.5056 (14)	142

Symmetry codes: (ii) −*x*+3/2, −*y*+1/2, *z*−1/2.
